# Israeli students’ perceptions regarding sperm donation: dilemmas reflections with dominant demographic effect

**DOI:** 10.1186/s12978-024-01767-4

**Published:** 2024-03-18

**Authors:** Itai Gat, Maya Ronen, Sarit Avraham, Michal Youngster, Ariel Hourvitz, Osnat Levtzion-Korach

**Affiliations:** 1 Sperm Bank & Andrology Unit, Shamir Medical Center, Zrifin, Israel; 2 IVF Department, Shamir Medical Center, Zrifin, Israel; 3 Faculty of Medical & Health Sciences, Tel Aviv University, Tel Aviv, Israel; 4 Shamir Medical Center, Zrifin, Israel; 5 Obstetrics and Gynecology Department, Shamir Medical Center, Zrifin, Israel

**Keywords:** Sperm donation, Sperm bank, Identity disclosure

## Abstract

**Background:**

Sperm donation has undergone significant medical and social transformations in recent decades. This study aimed to explore Israeli students’ perceptions towards sperm donation and investigate the potential influence of demographic characteristics on these perceptions.

**Design:**

The study encompassed 254 students from Tel-Aviv University, who completed an anonymous online survey in January–February 2021. This cross-sectional quantitative online survey, comprised 35 questions categorized into three sections: demographic data, assessment of prior knowledge, and perceptions of sperm donation (general perceptions related to both positive and negative stigmas associated with sperm donation, the roles and activities of sperm banks, and considerations surrounding identity disclosure versus the anonymity of sperm donors and their offspring).

**Results:**

Participants exhibited a relatively low level of prior knowledge (mean 31.2 ± 19 of 100). Scores for positive and negative stigmas ranged from 1.3 to 2.2. Notably, the statement “Donors’ anonymity preservation is crucial to maintain sperm donation” received a mean of 3.7. Seeking for anonymous sperm donation identity both by recipients and offspring was ranked with low means (1.5 and 1.7, respectively). However, the pursuit of half-siblings by mothers or siblings themselves received higher ratings ranging from 2.7 to 3. Women’s stigma ranking were notably lower, while men emphasized the importance of donor anonymity.

**Conclusions:**

Sperm Banks hold a position of medical authority rather than being perceived as being commercial entity. The preservation of donor anonymity is widely accepted as a crucial element, prioritized over the requests for identity disclosure from recipients and offspring. Demographic parameters exhibit a strong and precise effects on participants’ perceptions.

**Supplementary Information:**

The online version contains supplementary material available at 10.1186/s12978-024-01767-4.

## Background

Sperm donation has evolved significantly since its initial introduction, spanning many decades. Initially sperm donation primarily provided a discreet, anonymous source of frozen-thawed sperm for heterosexual couples grappling with male infertility [1, 2]. Donor selection in those early days was primarily rooted in attributes such as general appearance with minimal emphasis on comprehensive medical assessment. Infectious and genetic screening was seldom conducted [3, 4]. Couples were primarily focused on expeditiously achieving pregnancy with minimal investigative or evaluative measures. However, on the ensuing decades, advancements in fertility treatments for male infertility have led to a progressive reduction in the number of heterosexual couples seeking sperm donation. This transformation has been accompanied by profound sociological shifts, resulting in an increased demand for sperm donation among single women and same-sex couples [5]. Consequently, sperm donation has become an increasingly normalized and discussed subject, both in the broader society and within individual families [6].

Modern sperm banks face a complex array of challenges. Serving As intermediaries between donors on the one side and recipients and their offspring on the other, they must prioritize the well-being and interests of all parties involved. However, it is essential to acknowledge that sperm donation recipients are far from a homogenous group. Primarily, a significant discrepancy in interests emerges between newly arrived patients seeking sperm donation and mothers who already given birth. The former group seeks a diverse and extensive pool of available sperm donors to facilitate optimal matching based on criteria such as appearance and genetic compatibility. Conversely, mothers’ interest lie in restricting their chosen donor’s availability to other women, thereby minimizing the risk of unintentional unions among half-siblings [7]. Second, single patients, same-sex couples, and heterosexual couples exhibit distinct and unique characteristics [8, 9]. Third, and perhaps most critically, the expanding population of adolescents and young adults conceived through sperm donation prompts profound discussions regarding their right to access information about their biological fathers [10, 11]. This right, however, often collides with the donor’s interest in preserving anonymity [12, 13]. The advent of social media has further complicated the landscape, making the maintenance of anonymity increasingly challenging. Mothers and half-siblings actively seek their biological connections and the potential formations of unions with individuals who share the same donor. In this intricate and conflicting environment, sperm banks are tasked with the delicate responsibility of balancing the interests of each party while upholding stringent medical and ethical standards.

In addition to social and ethical dimensions, advances in genetic diagnostics and screening practices have assumed a significant role in the routine activities of sperm banks. in the past decade, the conventional ethnically based screening methodology [14] has faced challenges from expanded carrier screening panels—a novel approach, resulting in higher detection rate of recessive genetic disorders [15]. Nevertheless, the implications of these breakthroughs give rise to crucial ethical concerns concerning the interests of donors versus recipients [16] and raise questions about the traditional stance of excluding donors diagnosed as carriers of genetic diseases [17].

Amid the intricate interplay of evolving medical, ethical and social factors, the demand for sperm donation continues to surge [18]. While existing studies on perceptions towards sperm donation predominantly concentrate on the perceptions of sperm donors and recipients [19–22], It is imperative to acknowledge that sperm donation is no longer confined to specific population segments; it has become a widespread and pertinent phenomenon. Consequently, a broader research framework is necessary to engage new audiences and communities [13].

Considering the comprehensive impact of social medical trends on sperm bank activities in various fundamental aspects, adjustments to regulations and guidelines become necessary. However, implementing novel policies should take into consideration not only personnel involved throughout the process of sperm donation (such as physicians, donors and recipients etc.) but also perceptions of general population. In their comprehensive review, which included 33 articles, Hudson et al. emphasized the limited nature of knowledge regarding public understandings of and attitudes towards gamete donation [23]. A more recent survey revealed support for egg and sperm donation (78%), for IVF in single women (61%) and for same-sex female couples (64%) among more than 8500 responders from 6 European countries, which may reflect a trend towards openness and acceptance of gamete donation [24]. However, these wide accessed studies were focused on general public. We believe that young adults’ population, represented by students, is more relevant cohort to investigate within that general concept. The primary objective of this current research was to extend the scope of scientific investigation beyond the immediate stakeholders in sperm donation. Our focus was on young adults represented by university students, a demographic not directly impacted by sperm donation but potentially linked through their social and personal networks, thus warranting a closer examination. Furthermore, this study sought to explore the influence of general demographic characteristics on attitudes toward sperm donation. The research pursued two primary objectives: (1) To conduct a thorough and comprehensive investigation of perceptions pertaining to sperm donation. We evaluated not only general perceptions but also paid specific attention to significant conflicts and dilemmas; (2) To gauge the impact of specific demographic variables on these perceptions, shedding light on the role of demographics in shaping individual perceptions.

## Methods

### Study design and procedure

The study was based on a cross-sectional quantitative online survey.

It involved Israeli students at Tel-Aviv University, who participated by completing an anonymous digital questionnaire during the period of January to February 2021. To recruit participants, invitations to participate in the study were published on popular social media platforms, such as Facebook and WhatsApp. Specifically, we targeted closed groups including thousands of members affiliated with Tel-Aviv University. The study was administered by M.R., a medical student at the university who had access to these groups. M.R made three separate announcements during the specified period, each containing a concise statement outlining the study’s purpose and an exclusive digital link to access the questionnaire. Authorization from the group administrators wasn’t required to share the study, as it is a common practice among students to conduct research and recruit respondents using this platform. Out of the tens of thousands members who were exposed to the invitation to participate in the research, almost 971 initiated and over 250 completed response.

Participants who clicked on the provided link were immediately directed to the digital questionnaire, which was implemented using QuestionPro system—an established open-access software designed for such research applications. Prior to initiating the study, a notification was presented, informing participants that, by clicking the next button to access the questionnaire, they were providing informed consent to participate anonymously. Only after confirming this consent by clicking the subsequent button were participants officially enrolled in the study, gaining access to the complete questionnaire. Participants who answered less than 80% of the questions were excluded from the study.

The study was approved by the Ethics Committee of Tel-Aviv University (research proposal no. 0002615-1).

### Study questionnaire

The questionnaire comprised a total of thirty-five questions, organized into three primary sections:

1. Demographic data*:* this section included nine closed ended questions covering demographic characteristics including fundamental parameters related to the essence of the research such as marital and paternal status as well as religiosity. Additionally, participants were asked about prior familiarity with sperm donation and individuals involved, both donors and recipients.
2. Prior knowledge assessment: to evaluate participants’ prior knowledge regarding sperm donation, six multiple-choice questions were included in this section;
3. Perceptions of sperm donation: the central component of the questionnaire consisted of twenty items related to perceptions toward sperm donation. Participants were tasked with rating these items on a 1–5 Likert scale, where 1 indicated “strongly disagree”, 2 “disagree”, 3 “neutral opinion”, 4 “agree”, 5 “strongly agree”. This part was further divided into three subcomponents: (a) General attitudes phrased as solid stigmas (either positive or negative) to provoke prejudiced responses rather than “politically correct” answers; (b) Sperm banks roles and activities: this segment probed participants’ perspectives on the multifaceted roles of sperm banks, including medical, commercial, and sociological aspects, among others. (c) The final set of questions focused on the complex issue of identity disclosure versus the preservation of donor and offspring anonymity.

In their review of sperm donation perceptions, Van den Broeck et al. observed that 23 out of 25 questionnaires were specifically tailored for this topic without undergoing psychometrically validation [19]. Given the diverse questionnaires already existing in the scientific literature on sperm donation, and recognizing the practical challenges associated with statistically validating an entirely new questionnaire, our approach was to primarily draw from previously used questions [23, 25–29] including questions numbered 16–21, 26, 29–30. Our original additions were focused on the role of sperm banks (medical vs. commercial—q. 23–25) and its impact on donors’ selection (q. 27–28). Furthermore, we specified different aspects of donors’ anonymity vs. disclosure related to recipients and offspring (q. 31–35). While this topic stand in the focus of diverse studies, herein we insisted on detailed assessment of each perspective. We made specific additions and adaptations to align with the characteristics of the current study population. In addition to researchers’ comprehensive discussions until approving all included questions, Prior to the formal initiation of the study, the questionnaire was piloted with 20 non-anonymous students aged between 23 and 40. The primary objectives of this pre-test were to assess the digital platform’s usability, evaluate drop-out rates, and gauge the clarity of the questions. Based on the feedback and observations from the pre-test, several questions were rephrased, and minor adjustments were made to ensure that participants could complete the questionnaire within a reasonable time frame. Consequently, participants were expected to complete the questionnaire within an estimated time frame of approximately 6 min, aimed at mitigating the drop-out rates (Additional file 1). The pilot data wasn’t included in the final sample of the study.

### Statistical analysis

The demographic data gathered in the initial section of the questionnaire, which was initially descriptive, was subsequently employed for the statistical analysis detailed below. The second section concerning prior knowledge assessment comprising of informative multiple-choice questions was assessed based on the number of correct answers per participant. Subsequently, a total grade per participant was computed on a scale of 0 to 100 points, with each question contributing 16.67 points to the overall score.

The focus of this study was the third questionnaire section, comprising 20 perceptions and attitudes rated using the Likert scale. Initially, we provided an overview of the cohort’s responses to the three sub-sections, which encompassed general perceptions, sperm banks’ roles and activities, and attitudes concerning identity disclosure versus anonymity preservation. To assess the validity of the results, we employed Spearman correlation analysis, particularly for statements originally presented in contrasting or identical forms. Subsequently, we utilized the Mann–Whitney test to investigate the impact of the various demographic parameters on participants’ perceptions. These parameters included gender, personal acquaintance with sperm donation, paternity status, and religiosity. To the best of our knowledge, religiosity was the only parameter which was investigated previously [30]. Most parameters have not been investigated but we hypothesized they may have important contribution to participants’ perceptions. For the specific comparison of seeking half-siblings and disclosure of donor’s identity, the Wilcoxon test was applied. A statistically significant result was defined as p-value less than 0.05.

## Results

### Population demographic characteristics

The recruitment announcements were initially disseminated across 13 closed groups on Facebook and WhatsApp, specifically targeting students at  Tel-Aviv University. A total of 971 individuals responded to one of the three applications links and proceeded to access the digital questionnaire. Among these respondents, 373 initiated the questionnaire. Ultimately, 254 participants successfully completed at least 80% of the questions, resulting in a study inclusion rate of 68%. Non-completing participants exhibited variability in the stages at which they discontinued the survey. Therefore, it is challenging to identify a specific section of the survey that significantly influenced the dropout rate or completion status. Completers and non-completers did not differ on demographic variables.

The median age of the study participants was 27, with an age range spanning from 19 to 57 years. The majority of responders were female without children. Medical school was the most prominent institute (106 students, 41.7% of total cohort) compared to all other faculties (148, 58.2%). The demographic characteristics of the participants are outlined in Table 1.

**Table 1:** Participants’ demographic profile (N, %)

Gender N = 254	Female	157	61.8
	Male	97	38.2
Marital status N = 254	Single	177	69.7
	Married	75	29.5
	Divorced	2	0.8
Number of children N = 251	0	208	82.9
	1	19	7.6
	2	15	6.0
	3	9	3.6
Academic degree N = 252	BA	133	52.8
	MA	67	26.6
	PhD	30	11.9
	Basic studies	2	0.8
	Other	20	7.9
Religion N = 254	Jewish	245	96.5
	Non-Jewish	9	3.5
Religiosity N = 254	Secular	210	82.7
	Traditional	30	11.8
	Religious	14	5.5

### Prior knowledge assessment

Regarding previous acquaintance with sperm donation, 132 participants (51.7%) indicated no personal familiarity with individuals involved with sperm donation. For 88 participants (34.6%) their sole source of information on this topic was the media. Notably, 92 students (36.2%) reported being acquainted with women who had used sperm donation. Interestingly, 3 men (1.2%) disclosed they had previously donated sperm. Additionally, 14 students (5.5%) had contemplated sperm donation but ultimately decided against it, and 28 participants (11.2%) had personal acquaintances who were sperm donors.

In the subsequent section, participants encountered six informative multiple-choice questions. 8.3% participants correctly answered a single question, while 33.1%, 31.1%, and 18.1% responded accurately 2,3, and 4 questions. Respectively. Only 7% and 2% provided correct answers for 5 and all 6 questions, respectively. The mean grade per participant for this section was 31.2 ± 19 out of a total of 100 points.

### Participants’ perceptions towards sperm donation

The third and central component of the questionnaire was subdivided into three subsections: general perceptions towards sperm donation; sperm banks’ roles and activities; and attitudes regarding identity disclosure versus anonymity preservation containing 7, 6, and 7 phrases, respectively. Participants’ responses are described in Table 2.

**Table 2:** Participants’ perceptions towards SD (mean score on 1–5 Likert scale)

	1—Strongly disagree (n, %)	2—Disagree (n, %)	3—Neutral opinion (n, %)	4—Agree (n, %)	5—Strongly agree (n, %)	Mean	SD
A: General perceptions^a^							
Sperm donation is one of the most noble actions a man can do for others^+^	80, 32.5%	86, 35%	42, 16.2%	25, 10.1%	15, 6.1%	2.2	1.1
Sperm donation may have negative psychological impact on the offspring^−^	75, 30.6%	76, 31%	57, 23.2%	30, 12.2%	7, 2.8%	2.2	2.2
Sperm donation decreases the risk for offspring illness compared to spouse pregnancy^+^	95, 39.2%	49, 20.2%	65, 26.8%	27, 11.1%	6, 2.4%	2.1	1.1
Sperm donation impairs women desire for relationship and family with former sperm donor^−^	133, 54.7%	40, 16.4%	45, 18.5%	18, 7.4%	7, 2.8%	1.9	1.1
Fertility treatments increases parents’ love for their children^+^	140, 58.3%	41, 17%	31, 12.9%	18, 7.5%	10, 4.1%	1.8	1.1
Sperm donation contradicts my principles and/or faith^−^	201, 83%	21, 8.6%	13, 5.3%	4, 1.6%	3, 1.2%	1.3	0.7
Parents love their children less if they are not genetically identical to them^−^	196, 81.3%	27, 11.2%	10, 4.1%	8, 3.3%	0	1.3	0.7
B: Sperm banks’ roles and activities							
Sperm bank is a medical institute—its role is to extremely expand medical investigation for sperm donors in order to minimize offspring’s medical risk although it may decrease donors’ supply	10, 3.9%	20, 7.9%	51, 20.2%	99, 39.2%	72, 8.5%	3.8	1
Sperm bank is a commercial institute designed to sell sperm—it should perform minimal medical investigations (but still more than a romantic spouse) and supply wide range of sperm donors	124, 51.4%	61, 25.3%	32, 13.2%	22, 9.1%	2, 0.8%4	1.8	1
Social factors (such as live birth limitation) should be considered even in case of impaired supply	36, 14.3%	32, 12.7%	56, 22.3%	81, 32.2%	46, 18.3%	3.3	1.2
Religious factors should be considered during sperm donation	165, 67.6%	47, 19.2%	15, 6.1%	11, 4.1%	6, 2.4%	1.5	0.9
Sperm donor selection by the patient should be performed according to medical considerations only (such as genetic matching)	57, 23%	70, 28.3%	62, 5.1%	39, 15.7%	19, 7.6%	2.6	1.2
Sperm donor selection by the patient should be performed according to personal parameters (appearance, occupation, religiosity)	37, 14.8%	85, 34.1%	92, 36.9%	28, 11.2%	7, 2.8%	2.5	0.9
C: Identity disclosure vs. anonymity							
Donors’ anonymity preservation is crucial to maintain sperm donation	16, 6.3%	26, 10.3%	59, 23.4%	62, 24.6%	89, 35.3%	3.7	1.2
Donors should be offered to choose between anonymous vs. extra paid identity disclosure donation	120, 48.9%	39, 15.9%	40, 16.3%	34, 13.8%	12, 4.9%	2.1	1.2
Offspring are eligible to seek their half siblings only within sperm bank settings and donor’s consent	49, 19.9%	48, 19.5%	54, 21.9%	55, 22.3%	40, 16.2%1	3	1.3
Offspring are eligible to seek their half siblings by social media without donor’s consent	69, 24.2%	84, 29.4%	49, 17.1%	43, 15%	40, 14%	2.8	1
Offspring’s mothers are eligible to look for half siblings from the same sperm donor while maintaining his anonymity	80, 32.6%	36, 14.6%	45, 18.3%	49, 20%	35, 14.2%9	2.7	1.4
Offspring are eligible to seek their sperm donor opposed to his consent and their mother’s obligation	143, 60.3%	58, 24.4%	17, 7.1%	10, 4.2%	9, 3.8%	1.7	1
Offspring’s mothers are eligible to know donor’s identity although they have committed to maintain anonymity	174, 73.1%	38, 15.9%	14, 5.8%	4, 1.6%	8, 3.3%	1.5	0.9

1—strongly disagree; 2—disagree; 3—neutral opinion, 4—agree; 5—strongly agree

^a^Positive and negative perceptions are marked by + and −, respectively

Within the general perceptions division, participants’ scoring of both positive and negative perceptions were notably uniform and tended to be low. The highest scores, averaging 2.2, demonstrated to positive stigma “Sperm donation is one of the noblest actions a man can do for others”, which mirrored the negative perception that “Sperm donation may have a negative psychological impact on the offspring”. The lowest score of 1.3 was assigned to the statement “Sperm donation contradicts my principles and/or faith” (Table 2A).

The Following sub-section focused on sperm banks’ roles and activities. Firstly, two opposing questions aimed to assess whether participants perceived sperm banks primarily as medical or commercial institutions. Participants expressed a notably high level of agreement (mean 3.8) with the medical role, contrasting with a conflicting mean grade of 1.8 for the commercial role. Similar but opposing phrasing of these phrases enabled us to perform the Spearman correlation test. This test revealed a significant and opposing correlation (− 0.32, p < 0.001). Secondly, participants, on average, assigned higher scores to social considerations, particularly relating to offspring birth limitation, higher then to religious concerns. Thirdly, the sub-section also assessed the importance of medical characteristics versus personal characteristics of sperm donors during the donor selection process by the patients. These criteria were graded relatively similarly (Table 2B).

The third sub-section focused on disclosure versus anonymity of sperm donors and their offspring. When comparing anonymous donation to the option of identity disclosure, participants demonstrated a distinct preference for the former. Participants, on average, ranked “Donors’ anonymity preservation is crucial to maintain sperm donation” with a higher mean score of 3.7, in contrast to the mean score of 2.1 for “Donors should be offered to choose between anonymous versus extra paid identity disclosure donation”. In the same line, seeking anonymous sperm donation identity by recipients and offspring was ranked with low means (1.5 and 1.7, respectively). Conversely, the pursuit of connections with half-siblings by mothers or the siblings themselves garnered higher ratings, with mean scores ranging from 2.7 to 3 (Table 2C). Notably, the mean score for sentences related to seeking half-siblings was significantly higher than sentences related to donor’s identity disclosure (2.7 vs. 1.6, p < 0.001).

### Impact of demographic characteristics on participants’ perceptions

Upon completion of the general cohort’s perceptions examination, we performed various comparisons that took into account demographic characteristics, including gender, prior acquaintance with sperm donation, paternity, and religiosity. Due to the wide range of data and since the examination of each parameter resulted in specific findings, our data presentation herein primarily focuses on statistically significant results. Non-significant data are available upon request.

In our study, we examined 156 women and 96 men. The analysis of the general perceptions section revealed a noteworthy trend of significantly lower ranking of stigmas by women. Interestingly, gender-related significant differences were evident in all four negative stigmas but only in one third of the positive dogmas. No substantial differences emerged between women and men concerning questions related to sperm banks’ roles and activities. However, several significant differences were found in the disclosure versus anonymity section. Men consistently assigned higher importance to the preservation of donor anonymity. “Donors’ anonymity preservation is crucial to maintain sperm donation” got a high mean score of 4.1 compared to 3.5 among women (p < 0.00,1). Additionally, men rated the idea that “Offspring’s mothers are eligible to look for half-siblings from the same sperm donor while maintaining his anonymity” with a mean score of 2.2 in contrast to women who rated it at 3 (p < 0.001). Furthermore, men demonstrated a significant preference for offspring to seek their half-siblings officially through the sperm bank, contingent to donor’s approval, while women were more receptive to the concept of siblings connecting through social media without the donor’s consent (Table 3A).

**Table 3:** Gender, paternity, and religiosity related significant differences (mean score on 1–5 Likert scale)

A: Gender				
General perceptions^a^	Women N = 156	Men N = 96	Z-score	P
Sperm donation may have negative psychological impact on the offspring^−^	2.1	2.5	− 2.56	< 0.01
Sperm donation impairs women desire for relationship and family with former sperm donor^−^	1.8	2	− 1.9	< 0.045
Fertility treatments increases parents’ love for their children^+^	1.7	2.1	− 2.37	< 0.017
Sperm donation contradicts my principles and/or faith^−^	1.2	1.4	− 2.66	< 0.007
Parents love their children less if they are not genetically identical to them^−^	1.1	1.6	− 4.04	< 0.001
Sperm banks’ roles and activities—NA				
Identity disclosure vs. anonymity				
Donors’ anonymity preservation is crucial to maintain sperm donation	3.5	4.1	− 3.45	< 0.001
Offspring are eligible to seek their half siblings only within sperm bank settings and donor’s consent	2.7	3.3	− 3.2	< 0.001
Offspring are eligible to seek their half siblings by social media without donor’s consent	2.9	2.5	− 2	< 0.04
Offspring’s mothers are eligible to look for half siblings from the same sperm donor while maintaining his anonymity	3	2.2	− 4.1	< 0.001

B: Paternity				
General perceptions^a^	No children N = 206	Parents N = 42	Z-score	P
Fertility treatments increases parents’ love for their children^+^	1.9	1.6	− 2	< 0.039
Parents love their children less if they are not genetically identical to them^−^	1.3	1.1	− 2.4	< 0.015
Sperm banks’ roles and activities				
Social factors (such as live birth limitation) should be considered even in case of impaired supply	3.2	3.7	− 2	< 0.036
Religious factors should be considered during sperm donation	1.4	2.1	− 3.55	< 0.001

C: Religiosity				
General perceptions^a^	Non-secular N = 34	Secular N = 210	Z-score	P
Sperm donation contradicts my principles and/or faith^−^	1.6	1.2	− 1.99	< 0.046
Sperm banks’ roles and activities				
Religious factors should be considered during sperm donation	2.5	1.3	− 6.1	< 0.001

1—strongly disagree; 2—disagree; 3—neutral opinion, 4—agree; 5—strongly agree

^a^Positive and negative perceptions are marked by + and −, respectively

**Fig. 1:**
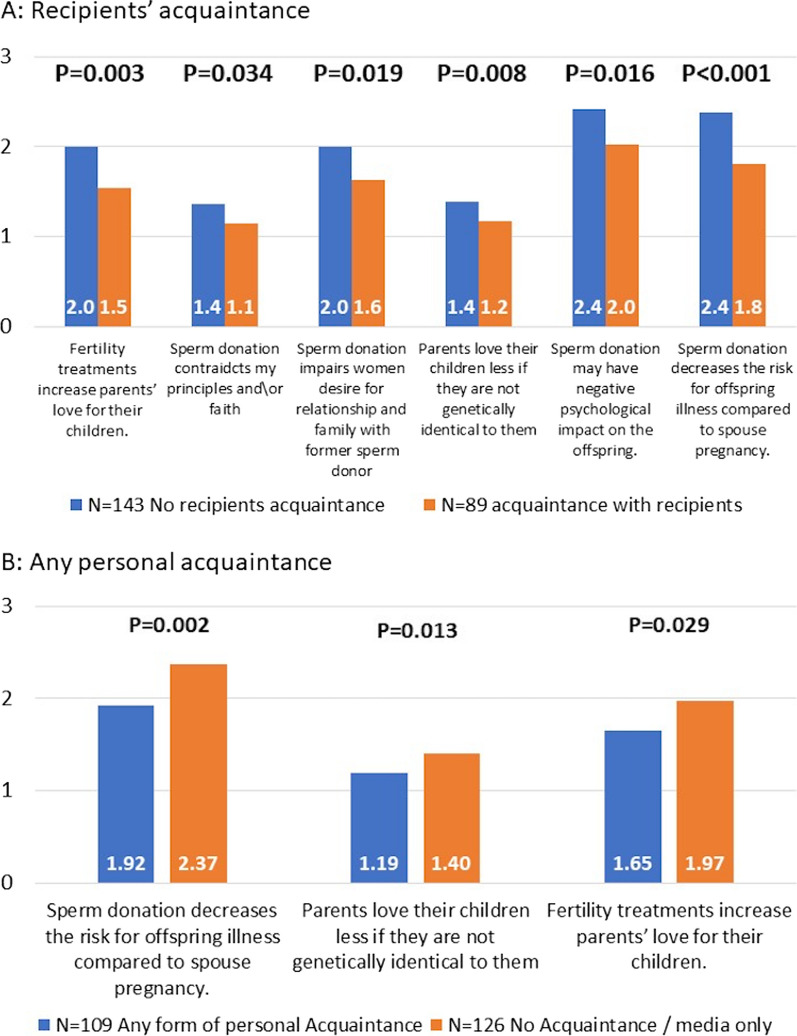
Acquaintance-related significant differences—the general perception

To explore the influence of prior acquaintance on participants’ perceptions, our investigation encompassed various categories: a personal history of sperm donation (for men); prior contemplation of sperm donation; familiarity with sperm donors, past reception of sperm donation (for women); acquaintance with recipients; acquaintance through media only, or no acquaintance at all. Notably, the most significant findings emerged among participants who were acquainted with sperm donation recipients (N = 92, 36.2%), particularly within the domain of general perceptions, six out of seven questions revealed significantly lower scores compared to other respondents. Participants lacking personal acquaintance (media only and no personal acquaintance, N = 132, 51.7%) ranked three out of seven viewpoints, both positive and negative, significantly higher than other respondents (Fig. 1).

In the comparisons made, no distinction surfaced in the evaluations regarding sperm banks’ role\activity and the aspects of anonymity versus disclosure. However, within a subset of respondents with a prior acquaintance with sperm donors (N = 28, 11%), notably higher agreement was observed concerning the statement “Offspring’s mothers are eligible to look for other offspring of their sperm donor while maintaining his anonymity” compared to other participants (3.37 vs 2.58, respectively, p = 0.009). No additional disparities in attitude were evident concerning prior acquaintance.

Paternity represents another significant demographic factor influencing respondents’ perceptions within specific clusters. Primarily, in the general perceptions section, both stigmas associated with the parent–child relationship received lower scores among parents (N = 42, 16.6%) compared to non-parents. Secondly, social, and religious considerations within sperm banks activity generated significantly higher scores compared to non-parents (Table 3B). No additional differences in perceptions related to paternity were evident.

Religiosity was specifically linked to two distinct statements: “Religious factors should be considered during sperm donation” and “Sperm donation contradicts my principles and/or faith” (related to general perceptions and sperm banks’ role and activity sections, respectively). Non-secular (religious and traditional) responders (N = 44, 17.3%) scored 1.34 versus 2.51 among secular responders for the first phrase (p < 0.0001) and 1.23 versus.1.6 (p = 0.046) for the second (Table 3C). No further variations in perceptions related to religiosity were discerned.

## Discussion

The influence of culture and religion on sexual and reproductive health and behaviour has increasingly become an area of study in contemporary time [31]. Sperm donation has evolved dramatically over the last decades, introducing a multitude of dilemmas. From a medical perspective, advancements in genetic technologies have opened new frontiers for the genetic assessment and diagnosis of donors, recipients, and offspring. Socially, there has been shift toward focusing on a healthy patient population rather than solely on the infertile male, altering the trajectory of medical treatment. The rights of offspring to know their biological father have led to regulatory changes in several countries, significantly impacting the population of sperm donors [32]. A considerable number of sperm donation programs offer open-identity sperm donation. In contrast to traditional anonymous donors, open-identity donors agree to release their identifying information to adult offspring [33]. Pacey et al. recently reported that more applicants are accepted as sperm donors among those who choose identity disclosure than those who prefer to maintain their anonymity [34]. As these trends are anticipated to progress, it becomes imperative to address previous regulations and concepts. Adapting policies and practices may offer better answer to developing and evolving demands. These evolving regulations should rely not only on professionals and participants in the sperm donation process but also consider the perceptions of the general population. We believe that such attitudes contribute to a global social viewpoint on sperm donation, demanding attention and thorough investigation.

To delve into these intricate matters, more than 250 students from diverse faculties responded to a targeted questionnaire. The findings represent herein reflect the perceptions of young adults—an important and relevant age group concerning sperm donation. Not surprisingly, nearly half of the participants reported a personal acquaintance with sperm donation. Yet, the relatively low objective knowledge score (with an average of 31/100) confirms our primary objective in recruiting participants who represent the general population rather than individuals already familiar with sperm donation. The terms “general population” or “public” are used to refer to those groups who have not necessarily had direct experience of either donating gametes or utilizing donated gametes in infertility treatment [23]. Furthermore, employing 6–7 questions for each section of the questionnaire enabled thorough investigation and assessment. Overall, participants’ responses to various questions exhibit consistent perceptions within each section, affirming the robustness of the findings presented.

### Participants’ perceptions regarding sperm donation

The questionnaire comprised seven solid stigmas, encompassing both positive and negative assertions. We adhered to extreme phrasing to trigger authentic responses (ex. “Parents love their children less if they are not genetically identical to them”). Participants’ responses reflected consistently low scores for both positive and negative stigmas (1.3–2.2). Decades ago, Edwards categorized diverse risks that preoccupy people in terms of gamete donation as psychological, biological, and relational [35]. However, growing and evolving data have unveiled a much more complex picture. Both adults and adolescents conceived by sperm donation have reported varied sentiments—ranging from ‘positive’ to ‘indifferent’ or ‘negative’—regarding their method of conception [18, 36]. Additionally, differences among recipients—comprising single women, same-sex couples and heterosexual couples—have been documented, further complicating the implications of sperm donation [37]. We interpret participants’ responses, which tend to diverge from stigmas, as indicative of a more nuanced perspective on sperm donation, transcending the simplistic notions of ‘good’ or ‘bad’. In other words, social trends observed among patients involved in sperm donation, are mirrored in public perceptions as well.

### Sperm banks’ roles and activities

The regulation of sperm banks activities is a matter of debate across countries and societies. While sperm donation for heterosexual couples is widely accepted and relatively common for single women, not all European countries permit sperm donation for female couples and, to an even lesser extent, for men couples [38]. The primary dilemma presented in the questionnaire was related to the fundamental conception of sperm banks—whether they should be viewed as medical or commercial institutions. According to Israeli regulations, each sperm bank must be associated with and operate under the affiliation of a medical centre, leading to a predominantly medical approach. Conversely, American sperm banks encompass both commercial and non-profit entities, operating within fertility centres or as stand-alone programs [33]. Remarkably, participants notably categorized sperm banks as medical rather than a commercial institutions. This clarification holds implications beyond theory. For instance, from ethical and legislative standpoints, sperm banks’ medical responsibility may limit patients’ autonomy, in contrast to the customer autonomy observed in commercial interactions.

Perspectives on sperm donation from both patients and physicians have yet to be fully ascertained and can be perceived across a spectrum. At one end of this spectrum, sperm banks may be regarded as a form of ‘romantic partner substitution’. Considering that most married couples typically do not undergo genetic screening for the male partner, some patients may perceive even basic evaluation of donors as supplementary and nonessential. Conversely, at the opposite end of this spectrum, sperm banks are seen as medical institutions bound by the obligation to utilize the most advanced technologies and methodologies. Participants in the present research assigned similar importance scores to both medical and non-medical factors throughout the donor selection process (scoring 2.6 and 2.5, respectively). We interpret this similarity as representing a balance or equilibrium on this matter.

While assessing medical and social impacts, we aimed to explore participants’ perceptions regarding a religious perspective. Schenker highlighted the importance of understanding diverse religious viewpoints concerning reproduction, as religious groups actively influence public bioethical stances, especially regarding procreation, abortion, and infertility treatment [39]. Notably, conservative monotheistic religions such as Judaism, Sunni Islam, and Roman Catholicism often impose limitations or even prohibit sperm donation [30, 38]. The statement ‘Religious factors should be considered during sperm donation’ received a low score of 1.6 in the current general cohort, primarily comprising secular participants. Nevertheless, significant differences emerged between religious and traditional participants compared to their secular counterparts. A possible implication of these findings relates to single religious women as a susceptible subgroup. Apart from the personal psychological burdens that might result from the absence of a romantic partner, they confront negative attitudes from their surroundings instead of receiving support while applying for sperm donation.

To summarize, while responders clearly defined sperm banks as medical institutions, recipients’ perspective is viewed as more complex. Social factors, such as restrictions on the number of offspring per donor, emerge as relevant and important.

### Sperm donors’ identity disclosure versus anonymity

There is a global trend toward open-identity gamete donation, with an increasing number of countries enacting legislation permitting only identifiable donors [40]. This trend aligns with the rising number of single women and same-sex couples seeking sperm donation, leading to heightened awareness concerning the long-term medical well-being and emotional welfare of offspring. Notably, female couples and single women pursuing donor insemination approach the prospect of potential contact with donor-linked families within a distinct relational context compared to heterosexual couples [37].

Despite the global trend advocating for donors’ identity disclosure, anonymity remains crucial for a significant portion of men who consider sperm donation [12]. Certain studies have highlighted adaptations in donors’ characteristics, including older age and having children of their own [41]. Other studies have underscored a noticeable shortage of donors to meet the growing demands, resulting in a rise of importation of sperm or reproduction traveling [42–44]. Recently, we reported that losing anonymity is the leading cause to avoid sperm donation among young students, who represent a potential population for donors’ recruitment [13]. Within the current cohort, a clear preference for donors’ anonymity over disclosure was evident. Firstly, the statement ‘Donors’ anonymity preservation is crucial to maintain sperm donation’ received a high score of 3.7. Secondly, the mean score of phrases associated with donor identity disclosure was significantly lower than those related to seeking half-siblings. These responses suggest a bidirectional perspective on this matter. On the one hand, students’ responses align with recipients’ expressed interest in connection with individuals sharing the same donor, as previously outlined [45]. Such desires are mainly relevant for single women and lesbians who seek the option of open-identity donation for their children [33]. On the other hand, participants expressed a significantly higher preference for maintaining donors’ anonymity. We posit that these findings, which oppose the overall global trend, may be explained through different perspectives. Firstly, sperm donation in Israel involves single men, which is contrasts with the open donor profile seen in other countries. Secondly, these findings might mirror ethical and psychosocial concerns associated with removal of anonymity. For instance, the decision to disclose conception details to offspring places substantial pressure on parents. Specifically, fathers may feel threatened by the donor, the potential impact of the disclosure on their relationship with their offspring, and the anonymity factor acts as a shield against the stigma linked to male infertility [10].

### Perceptions’ selectivity related to demographic parameters

A notable finding from the current research involves the substantial influence of demographic characteristics on participants’ perceptions (Table 3). Particularly noteworthy was the impact of gender, with women exhibiting a greater level of acceptance toward sperm donation, displaying higher willingness for identity disclosure, either in seeking half-siblings or revealing donors’ identities, compared to men. Another significant observation was that personal acquaintance with patients or any involvement in sperm donation resulted in lower stigma scores. This may reflect former prejudice, which is confronted and challenged in case of personal exposure. While difference related to religiosity have been previously reported [30], studies focusing on the general population are relatively scarce. To the best of our knowledge, this is the first extensive study focusing on the student age group that unveils perceptions selectively linked to demographics. Therefore, our hypothesis regarding the importance of these parameters was confirmed.

### Importance of current findings and “real world” possible applications

An important aspect of the current research lies in broadening the scientific inquiry beyond the scope of sperm donors and recipients to encompass young adults who are not directly involved in sperm donation but share common demographic characteristics such as age and marital status. The current cohort demonstrates a rather surprising level of comprehension regarding the contentious issues associated with sperm donation.

These findings align with several ongoing processes that have evolved over recent decades. Firstly, expanding genetic screening for donors, aimed to reduce the risk for hereditary disease among offspring, is supported by our participants despite the possible restriction impact on donors’ supply. Secondly, donors’ anonymity is strongly upheld as a fundamental and crucial principle in sustaining sperm donation. Israeli regulations, which exclusively permit anonymous donations are widely accepted among the participants. Thirdly, our study demonstrated a different attitude towards recipients versus offspring related to seeking relatives. These results suggest the potential merit in establishing a formal system, possibly coordinated by sperm banks, that would facilitate offspring in finding their half-siblings while upholding the anonymity of the donor.

### Limitations

The current research possesses several limitations that merit acknowledgment. Primarily, the focus on a student population may not offer a wholly reliable representation of the entire general populace. However, our study aimed to concentrate on the perceptions toward sperm donation among young adults, making this limitation justifiable. Additionally, the student population may not precisely mirror the young adult segment of the general population. The inclusion of participants without children holds relevance for the study, as it allows for an exploration of perceptions among those who have stronger linkage to the process of sperm donation. In the contrary, the dominant impact of women (more than 60%) and medical students (41.6%) require additional studies with different cohorts (such as parents, religious participants etc.) to confirm our results. Conducting investigations within the broader general population necessitates different settings, which fall outside the scope of the present study. Nevertheless, by concentrating on students, we facilitated a detailed examination using specific inquiries (e.g., those focused on donors’ anonymity versus disclosure), leading to a comprehensive and statistically significant outcome. Despite the accepted usage of non-validated questionnaires in the literature, the lack of psychometric validation in the current questionnaire is another limitation. Developing specific validated tool for the sake of sperm banks related studies is challenging. Yet, future efforts should be made to establish reliable and validated questionnaire, which may be used by diverse studies in various populations to improve our scientific methodology.

## Conclusions

The current research demonstrates several important findings, including the general acceptance of sperm donation, the recognition of sperm banks as medical institutions rather than commercial entities, a high priority placed on preserving donors’ anonymity alongside tolerance towards seeking half-siblings, and the substantial influence of demographic parameters on participant’ perceptions.

The research boasts two primary strengths: firstly, its large sample size allowed a comprehensive examination, revealing significant outcomes even in minor numerical differences. Secondly, the research questionnaire incorporated various statements in each component, leading to consistently significant results across different questions. This consistency lends high validation to the presented findings. Given that most literature focuses on either sperm donors or recipients, this study offers diverse perspectives within a relevant age group. The current findings should be taken into account by policymakers and committee opinions when establishing adaptations in sperm donation practices. While differences between heterosexual couples versus single women and lesbian couples have been extensively explored, attention should also be directed towards conservative and religious patients seeking sperm donation. The importance of demographic characteristics opens new optional direction for future studies such as focusing on the impact of socioeconomic status and ethnical background regarding sperm donation among the public, recipients, and donors.

### Supplementary Information

**Additional file 1.** Research questionnaire.

## Data Availability

The study’s data is available upon request.
