# Supplementary material for: Israeli students’ perceptions regarding sperm donation: dilemmas reflections with dominant demographic effect
Source: Reprod Health. 2024 Mar 18;21:37. doi: 10.1186/s12978-024-01767-4 (PMC10946193; doi:10.1186/s12978-024-01767-4)
Supplement: Supplementary file 1 — Additional file 1. Research questionnaire. [file 12978_2024_1767_MOESM1_ESM.docx]

**Additional file 1: Research questionnaire**

**Demographic data**

1. Gender.
2. Age.
3. Marital status.
4. Children.
5. Education.
6. University faculty.
7. Religiosity.
8. Socioeconomic status.
9. What is your personal acquaintance with sperm donation?

**Previous knowledge regarding sperm donation** (corrected answers are in **bold**):

1. How many sperm banks exist in Israel? (1-5 \ 6-10 \ 11-15 \ **>15**)
2. How many male applicants are eventually accepted to become sperm donors? **(<20%** \ 20-40% \ 40-60% \ > 60%)
3. How long does it take (in months) to complete the medical evaluation of a sperm donation candidate, starting from his initial application until final approval as a sperm donor? (>1 \ 1-3 \ 3-5 \ **5 <**)
4. Only anonymous donations are allowed in Israel. (right \ **wrong)**
5. Sperm donation is Israel is permitted from local donors only. (**right** \ wrong)
6. Which is the main population who applies for sperm donation? **(Single women** \ same sex couples \ heterosexual couples)

**Perceptions and attitudes towards sperm donation**

General perceptions

1. **Sperm donation is one of the most noble actions a man can do for others.**
2. **Sperm donation contradicts my principles and\or faith.**
3. **Sperm donation impairs women desire for relationship and family with former sperm donor.**
4. **Parents love their children less if they are not genetically identical to them.**
5. **Fertility treatments increase parents’ love for their children.**
6. **Sperm donation may have negative psychological impact on the offspring.**
7. Sperm donation decreases the risk for offspring illness compared to spouse pregnancy.

Sperm banks' roles & activities

1. Sperm bank is a medical factor - its role is to extremely expand medical investigation for sperm donors in order to minimize offspring's medical risk although it may decrease donors' supply.
2. Sperm bank is a commercial factor designed to sell sperm - it should perform minimal medical investigations (but still more than a romantic spouse) and supply wide range of sperm donors.
3. Social factors (such as live birth limitation) should be considered even in case of impaired supply.
4. **Religious factors should be considered during sperm donation.**
5. Sperm donor selection by the patient should be performed according to medical considerations only (such as genetic matching).
6. Sperm donor selection by the patient should be performed according to personal parameters (appearance, occupation, religiosity).

Identity disclosure vs. anonymity

1. **Donors' anonymity preservation is crucial to maintain sperm donation.**
2. **Donors should be offered to choose between anonymous vs. extra paid identity disclosure donation.**
3. Offspring's mothers are eligible to know donor's identity although they have committed to maintain anonymity.
4. Offspring's mothers are eligible to look for half siblings from the same sperm donor while maintaining his anonymity.
5. Offspring are eligible to seek their sperm donor opposed to his consent and their mother’s obligation.
6. Offspring are eligible to seek their half siblings by social media without donor's consent.
7. Offspring are eligible to seek their half siblings only within sperm bank settings and donor's consent.
